# Salivary nitrate prevents osteoporosis via regulating bone marrow mesenchymal stem cells proliferation and differentiation

**DOI:** 10.1016/j.jot.2023.12.001

**Published:** 2024-03-25

**Authors:** Xiaoyu Li, Lei Hu, Xue Wang, Huan Liu, Chunmei Zhang, Jinsong Wang, Xiaogang Wang, Songlin Wang

**Affiliations:** a Salivary Gland Disease Center and Beijing Key Laboratory of Tooth Regeneration and Function Reconstruction, Beijing Laboratory of Oral Health and Beijing Stomatological Hospital, Capital Medical University, Beijing, 100050, China; b Department of Biochemistry and Molecular Biology, Capital Medical University School of Basic Medicine, Beijing, 100069, China; c Key Laboratory of Big Data-Based Precision Medicine, School of Engineering Medicine, Beihang University, Beijing, 100191, China; d Immunology Research Center for Oral and Systemic Health, Beijing Friendship Hospital, Capital Medical University, Beijing, 100050, China; e Laboratory of Homeostasic Medicine, School of Medicine, Southern University of Science and Technology, Shenzhen, 518055, China; f Research Unit of Tooth Development and Regeneration, Chinese Academy of Medical Sciences, Beijing, 100700, China

**Keywords:** Bone marrow mesenchymal stem cells, EGFR, mTOR, Nitrate, Osteoporosis, Salivary

## Abstract

**Background:**

Nitrate, a key component of saliva, has been shown widely physiological functions in the human body. But its function on bone metabolism remains unclear. The aim of this study was to investigate the function and mechanism of saliva nitrate on osteoporosis and the function of bone marrow mesenchymal stem cells (BMSCs).

**Methods:**

Saliva nitrate removal or supplemental interventions were performed for 1 month in ovariectomized (OVX) osteopenia mice. The nitrate levels in saliva and serum were detected. The bone formation and bone microarchitecture in the OVX mouse model were investigated by quantitative Micro--computed tomography imaging, histological staining and serum bone biomarker analysis. The effects of nitrate on the functional homeostasis of BMSCs in OVX mice were explored by Ki67 immunofluorescence staining, Ki67 flow staining, alizarin red staining, qPCR and western blotting. Finally, downstream signaling pathways were screened by proteomics and verified by western blotting.

**Results:**

The results showed that nitrate deficiency exacerbated osteoporosis, while nitrate administration prevent osteoporosis in OVX mice. Nitrate increased the expression of PINP, a biomarker of bone formation, in OVX mice. Besides, nitrate enhanced the proliferative capacity and osteogenic function of BMSCs in OVX mice *in vitro* and *in vivo*. In addition, nitrate upregulated the expression levels of osteogenesis-related genes *ALP*, *Run2* and *OPN* of BMSCs. EGFR and mTOR signaling were screened as the key downstream of nitrate, and phosphorylated protein levels of its subfamily members AKT, ERK and S6K were significantly upregulated by nitrate.

**Conclusion:**

The present results showed saliva nitrate preventively protects against osteoporosis through enhances the proliferation and osteogenic differentiation potential of BMSCs. The effects of nitrate on bone homeostasis are closely related to the EGFR/AKT/ERK and mTOR/S6K signaling axes.

**The translational potential of this article:**

Our study provides experimental evidence for the use of saliva nitrate as an effective candidate for the prevention of osteoporosis and maintenance of bone homeostasis.

## Graphical abstract

**Figure:**
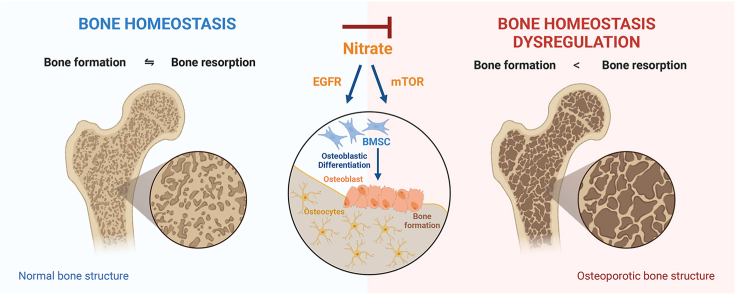

## 1 Introduction

Osteoporosis is a systemic metabolic bone disease characterized by decreased bone mass, reduced bone density, altered bone microstructure and increased bone fragility [1]. The prevalence of osteoporosis is increasing every year with the aging population [2]. As a result, osteoporosis and the associated risk of fragility fractures have become a major disease of concern worldwide, seriously affecting people's health [3,4]. Bone marrow mesenchymal stem cells (BMSCs) have self-renewal and multidirectional differentiation potential, and play an important role in maintaining the stability of the bone marrow internal environment [[5], [6], [7]]. During the development of osteoporosis, the balance is disturbed by the decreased ability of BMSCs to differentiate into osteoblasts and the increased ability to differentiate into adipocytes, which leads to disturbances in bone metabolism and ultimately to disease progression [8,9]. Therefore, it is scientifically important to further investigate the methodological means by which the functional homeostasis of BMSCs can be regulated in order to develop new therapies for osteoporosis and maintain bone homeostasis.

It is well known that organ–organ communication could regulate the bone metabolism, like liver, intestine et al. [10]. Decades ago, evidence have showed dysfunction of salivary gland have a tight relationship with osteoporosis, but the mechanism remains unclear [11]. Saliva is secreta by salivary gland, with contains variant components, like Na, K, Cl, amylase, nitrate et al. [12]. Inorganic nitrate is a natural component in our diet, especially in green leafy vegetables. Nitrate ingested via the oral cavity is absorbed into the blood in the gastrointestinal tract and enters the systemic circulation, and approximately 25 % of the nitrate in the blood is uptaken and transported to the saliva via the salivary glands, resulting in a nitrate concentration in the saliva that is approximately 10 times the level in the blood [13]. Salivary nitrate achieves prevention of intestinal inflammatory reactions by regulating the balance of intestinal flora [14], prevention of systemic radiation damage by lowering the level of reactive oxygen species [15], protection of salivary gland function by inhibition of apoptosis [16], and prevention of hepatic senescence [17]. Furthermore, in metabolic disorders, long-term nitrate-reduced diets have led to a range of metabolic abnormalities in C57BL/6J mice, including increased visceral obesity, dyslipidemia, decreased glucose tolerance, and insulin resistance [18]. There is also evidence that inorganic nitrate promotes the conversion of white adipose tissue to brown adipose tissue by activating the nitrate-nitrite-NO pathway [19]. However, osteoporosis as a systemic metabolic bone disease, whether nitrates can be used as a therapeutic agent for osteoporosis by altering bone metabolism is still contradictory in current findings.

Therefore, in this study, we aimed to explore the role and mechanism of salivary nitrate in regulating the function of BMSCs and thus preventing osteoporosis. Our findings suggest that nitrate enhances the osteogenic differentiation potential of BMSCs by regulating EGFR and mTOR signaling pathways, thereby preventing the osteoporosis.

## 2 Materials and methods

### 2.1 Mice

This study was approved by the Animal Care and Use Committee of Capital Medical University. Female C57BL/6 mice (8 weeks of age), purchased from Beijing Vital River Laboratory Animal (Beijing, China), were randomly divided into four groups (n = 6/group), including the Sham group (Sham operation), the OVX group (bilateral ovariectomy, bilateral OVX), the de-nitrate group (bilateral OVX and removal of salivary nitrates), and the 4 mM nitrate group (bilateral OVX and administration of 4 mM nitrate). Nitrate removers (JBL, Germany) were placed in the mouths of mice and changed weekly. Inorganic nitrate (Sigma–Aldrich, USA) was dissolved in drinking water for administration. Mice were maintained in a specific pathogen-free animal facility and kept under conventional conditions with free access to water and food. One month after operation, all mice were killed and samples from each group were collected.

### 2.2 Nitrate level determination

Saliva and serum of mice from each group were filtered and diluted by 10,000 MW. The concentration of nitrate was detected with Total Nitric Oxide and Nitrate/Nitrite Parameter Assay Kit (KGE001, R&D, USA), following standard experimental procedures provided by the manufacturer.

### 2.3 Enzyme-linked immunosorbent (ELISA) assay

The levels of the bone formation marker procollagen type I N-terminal propeptide (PINP) in the serum of mice from each group were measured by ELISA kits (E-EL-H0185c, Elabscience, China) according to the manufacturer's instructions.

### 2.4 Micro-computed tomography (micro-CT) analysis

The femoral bone structure of mice was analyzed using a high-resolution Inveon CT/PET/SPECT scanner (Siemens, German). Two- and three-dimensional bone structure image slices were reconstructed and scan data were analyzed using CT-Analyser, CT-Volume and CT-Voxel software (Skyscan, Belgium).

### 2.5 Histological analyses

The femurs of mice were fixed with 4 % paraformaldehyde (PFA) for 2 days and then decalcified in 10 % EDTA for 2 months. Sections (5-μm) were prepared, stained with hematoxylin and eosin (H&E) and Masson's trichrome (Maxim Biotechnologies, China) according to the manufacturer's protocol.

### 2.6 Cell culture of BMSCs

BMSCs were cultured from bone cavity of femurs and tibias of C57BL/6 mice. The cells were cultured with alpha-MEM medium (Gibco, USA) supplemented with 20 % fetal bovine serum (FBS, Gibco), 2-mM glutamine, 100-U/mL penicillin, and 100-mg/mL streptomycin (Invitrogen, USA), and then incubated in 5 % carbon dioxide at 37 °C. BMSCs between passages 3–4 was used in the following study.

### 2.7 Flow cytometric analysis

Bone marrow cells in bone cavity of the femur and tibia of mice were washed out with PBS containing 3 % FBS and treated with Red Blood Cell Lysis Buffer (R1010, Solarbio, China). Intranuclear staining was performed with Fixation/Permeabilization Buffer (00-5521-00, eBioscience, USA) according to the manufacturer's instructions. Anti-mouse-CD105-APC, -Sca-1-Pacific Blue, -CD45-PE, -CD11b-PerCP and -CD90-FITC were used for stem cell surface staining to circle the cell population of BMSCs. Anti-mouse-Ki67-PE-eFluor 610 was used to circle the Ki67-positive cell population in BMSCs. Stained cells were analyzed on FACSymphony (BD Biosciences, USA) and data were analyzed with FlowJo software (BD Biosciences).

### 2.8 Osteogenic differentiation detection

BMSCs were cultured with osteogenic inductive medium (MUBMX-01001, Cyagen, China). After 21 days, the mineralized nodules were stained with 2 % Alizarin Red (A5533, Sigma–Aldrich). After solubilizing in 10 % cetylpyridinium chloride (8,400,080,025, CPC, Sigma–Aldrich) for 30 min at room temperature, areas of the Alizarin red stain were measured by the microplate reader at a wavelength of 560 nm.

### 2.9 Immunofluorescence staining

BMSCs were fixed with 4 % PFA for 15 min and permeabilized with 0.5 % Triton X-100 for 20 min at room temperature. Then cells were blocked with normal goat serum for 30 min, and finally incubated with rabbit polyclonal anti-ki67 (1:200, ab15580, Abcam, USA) overnight at 4 °C. The next day, cells were incubated with donkey anti-rabbit IgG (H + L) Alexa Fluor 594 (1:1,000, A-21207, Invitrogen, USA) for 1 h at room temperature before counterstaining the nuclei with DAPI (Invitrogen). IF images were taken using the confocal microscopy (Leica, Germany).

### 2.10 RT-PCR analysis

Total RNA was isolated from BMSCs using the RNAios Plus reagent (TaKaRa, Japan) and was reverse transcribed to cDNA using the PrimeScript TM RT Reagent Kit with gDNA Eraser (TaKaRa). Target genes were amplified with cDNA, specific primers and NovoStart®SYBR qPCR SuperMix plus (Novoprotein, China) in a CFX96 Touch Real-Time PCR detection system (Bio-Rad Laboratories, USA). PCR amplification was performed in triplicate at 95 °C for 15 s, followed by 40 cycles at 95 °C for 5 s, at 60 °C for 30 s, at 95 °C for 15 s, at 60 °C for 15 s, and at 95 °C for 15 s. The relative expression levels of the target gene were normalized to GAPDH levels and determined by the 2^−ΔΔCt^ method. The information on the primers is shown in Table S1 (Supplemental file 1).

### 2.11 Western blot analysis

BMSCs were lysed using RIPA reagent containing 1 % PMSF and 1 % phosphatase inhibitor cocktail. After centrifugation at 14,000 g for 5 min, total protein concentrations were measured using a BCA Protein Assay Kit (PC0020, Solarbio). Proteins were then separated by sodium dodecyl sulphate–polyacrylamide gel electrophoresis (SDS-PAGE) and transferred onto polyvinylidene difluoride (PVDF) membranes (Millipore, USA). The protein bands were then developed with the use of the Pierce ECL Western Blotting Substrate (32,209, Thermo Fisher Scientific, USA), and the densitometry of each band was conducted using ImageJ (National Institutes of Health). The following primary antibodies were used: pEGFR (1:1,000, #3777S, CST), EGFR (1:1,000, #4267S, CST), pERK (1:1,000, #4370S, CST), ERK (1:1,000, #4695S, CST), pAKT (1:1,000, #4060S, CST), AKT (1:1,000, #4691S, CST), pS6K (1:1,000, #9234S, CST), S6K (1:1,000, #34475S, CST), GAPDH (1:1,000, ab8245, Abcam).

### 2.12 Proteomics analysis

The RayBio® Human Phosphorylation Pathway Assay Array C55 simultaneously detects the relative phosphorylation levels of 55 unique proteins in five well-known signaling pathways: MAPK, AKT, JAK/STAT, NF-κB, and TGF-β. Each antibody is carefully validated using appropriate cell lysates. The data were extracted using the instrument's own analysis software, and the data analysis software of AAH-PPP-1 was used to perform data pre-analysis.

### 2.13 Statistical analysis

All experiments are randomized into groups of similar sample size by block randomization. Data collection and analysis were performed blindly. All the experiments were performed independently at least three times, and data were expressed as mean ± SD. For a comparison of only two groups, we used Student's *t*-test. The statistical analyses were conducted by Prism (GraphPad Prism v7.02), and the values of p < 0.05 were considered to be statistically significant.

## 3 Results

### 3.1 Removal of salivary nitrate reduced nitrate concentration in saliva and serum of OVX mice

**Fig. 1:**
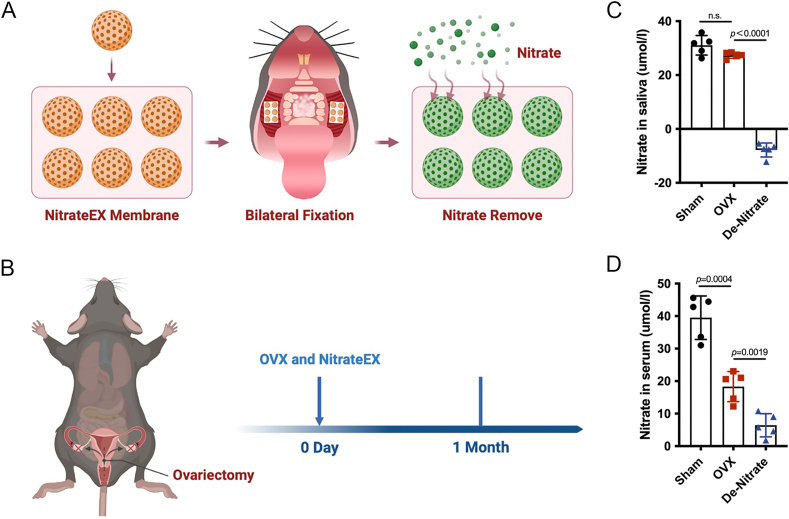
**Construction of nitrate removal in OVX mice.** A, Schematic diagram of removing nitrate from mouse saliva. B, Surgical pattern diagram. C, Nitrate was successfully removed from the saliva of mice in the de-nitrate group. D, Nitrate concentration in the serum of mice in the de-nitrate group was the lowest. Data are presented as mean ± SD, n = 5 (n.s., no significance).

To investigate the effect of salivary nitrate on osteoporosis, we used nitrate remover to remove salivary nitrate in OVX mice while constructing the OVX mouse model, and observed the bone quality changes one month later (Fig. 1A and B). Nitrate concentration in saliva of OVX group was lower than that of Sham group, but there was no statistically significant difference. Meanwhile, nitrate concentration in saliva of de-nitrate group was not detected and was significantly lower than that of OVX group (Fig. 1C). Nitrate concentration in serum of OVX group was lower than that of Sham group, while nitrate concentration in serum of de-nitrate group was even lower than that of OVX group (Fig. 1D). The above results indicated that the nitrate content in saliva and serum would be reduced when OVX mice caused osteoporosis, and OVX mice with removal of salivary nitrate was successfully constructed.

### 3.2 Salivary nitrate removal aggravated osteoporosis in OVX mice

**Fig. 2:**
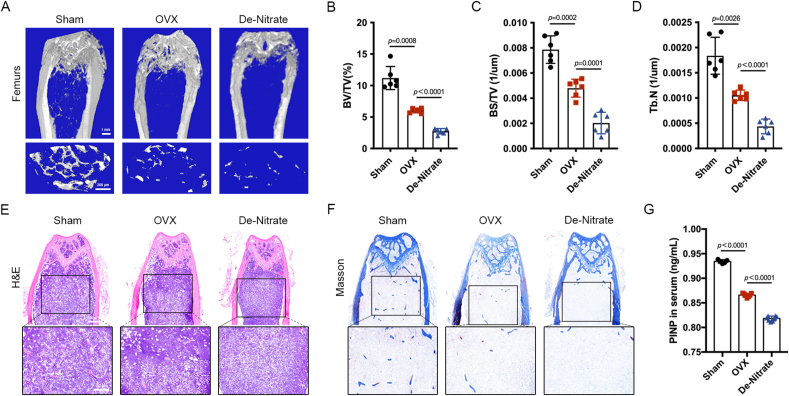
**Nitrate removal aggravated osteoporosis in OVX mice.** A, Micro-CT results. B-D, The bone histomorphology-related indexes BV/TV, BS/TV and Tb.N in the femur of OVX group were lower than those of Sham group, and the de-nitrate group was even lower than the OVX group. E, H&E staining. F, Masson staining. G, PINP level in the de-nitrate group was the lowest. Data are presented as mean ± SD, n = 6.

The micro-CT images of femoral trabecular structures were shown in Fig. 2A. The bone histomorphometic analysis demonstrated that compared to those of Sham group, the bone volume fraction (BV/TV), bone surface area bone volume ratio (BS/TV) and bone trabeculae number (Tb.N) of femurs in the de-nitrate group were reduced (Fig. 2B–D), indicating that the osteoporosis modeling was successful. These indices were lower in the de-nitrate group compared with the OVX group, indicating that bone loss was more severe in the de-nitrate group than in the OVX group (Fig. 2B–D). The results of H&E staining and Masson staining showed that the mineralization level of femur bone was significantly lower in the OVX group compared with the Sham group, and the mice in the de-nitrate group were even lower than the OVX group (Fig. 2E and F). In addition, PINP, a marker of bone formation, was significantly lower in the serum of OVX group compared with Sham group, and was even lower in the serum of de-nitrate group compared with OVX group (Fig. 2G), indicating that the osteoblast activity and bone formation status of de-nitrate group were worse. Taken together, these results suggested that salivary nitrate removal exacerbated the severity of osteoporosis in OVX mice.

### 3.3 Nitrate supplementation significantly increased nitrate concentration in saliva and serum of OVX mice

**Fig. 3:**
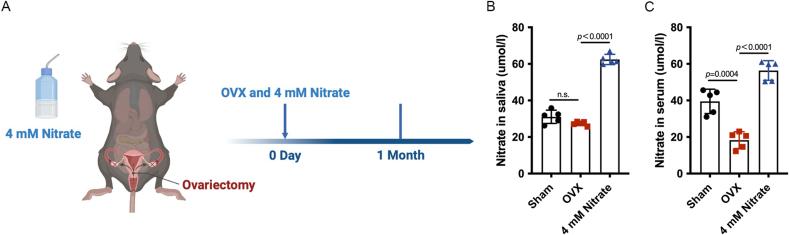
**Construction of nitrate-supplemented OVX mice.** A, Surgical pattern diagram. B, Nitrate was successfully increased from the saliva of mice in the 4 mM Nitrate group. C, Nitrate concentration in the serum of mice in the 4 mM Nitrate group was increased. Data are presented as mean ± SD, n = 5 (n.s., no significance).

In order to further determine whether nitrate has a preventive effect on osteoporosis, the OVX mice were constructed while 4 mM nitrate was given in water, and the phenotypic changes of mice were observed after one month (Fig. 3A). The nitrate concentration in the saliva of OVX group was lower than that of Sham group, but there was no statistically significant difference, and the administration of 4 mM nitrate significantly increased the nitrate concentration in the saliva of the OVX mice (Fig. 3B). The nitrate concentration in the serum of OVX group was lower than that of Sham group, but the administration of 4 mM nitrate significantly increased the nitrate concentration in the serum of the OVX mice (Fig. 3C). The above results indicated that the nitrate supplemented OVX mice was successfully constructed.

### 3.4 Nitrate supplementation prevents osteoporosis in OVX mice

**Fig. 4:**
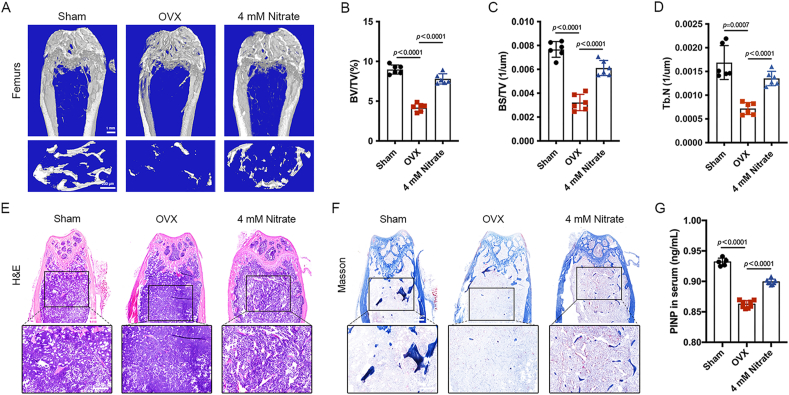
**Nitrate supplementation prevented osteoporosis in OVX mice.** A, Micro-CT results. B-D, The bone histomorphology-related indexes BV/TV, BS/TV and Tb.N in the femur of OVX group were lower than those of Sham group, while nitrate supplementation significantly improved these indices in the OVX mice. E, H&E staining. F, Masson staining. G, Nitrate supplementation increased PINP levels in OVX mice. Data are presented as mean ± SD, n = 6.

The micro-CT images of femoral trabecular structures were shown in Fig. 4A. Analysis of bone histomorphometric indexes showed that BV/TV, BS/TV, and Tb.N of femurs in the OVX group were lower than those in the Sham group, which indicated that the osteoporosis model was successfully modeled (Fig. 4B–D). However, the above indexes were significantly higher in the femurs of 4 mM Nitrate group compared with OVX group, which indicated that the bone volume of mice in the Nitrate supplemented could improve the bone mass of OVX mice (Fig. 4B–D). The results of H&E staining and Masson staining showed that the mineralization level of the femur in the OVX group was significantly lower than that in the Sham group, while supplementation with 4 mM nitrate increased the mineralization level of the femur in OVX mice (Fig. 4E and F). In addition, PINP in the serum of OVX group was significantly lower compared to Sham group, but PINP in the blood of 4 mM Nitrate group was higher than that of OVX group (Fig. 4G), reflecting better osteoblast activity and bone formation status in the 4 mM Nitrate group. These results suggested that nitrate supplementation prevented the progression of osteoporosis in OVX mice.

### 3.5 Nitrate enhanced the proliferation and osteogenic differentiation potential of BMSCs in OVX mice

**Fig. 5:**
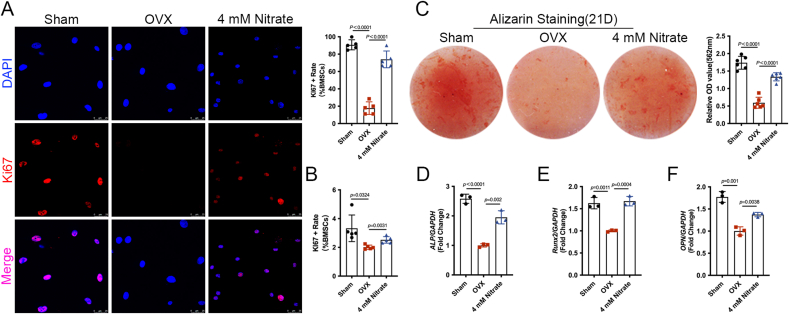
**Nitrate enhanced the proliferation and osteogenic differentiation potential of BMSCs in OVX mice.** A, Immunofluorescence staining of Ki67 (red) and DAPI (blue) of BMSCs. Quantitative analysis indicated significantly more Ki67-positive cells in 4 mM Nitrate group compared with the OVX group. B, Flow cytometry analysis showed nitrate supplementation increased Ki67-positive cells in BMSCs of the OVX group. C, Alizarin Red staining and quantitative analysis indicated that more mineralized nodules in 4 mM Nitrate group compared with the OVX group. D-F, RT-PCR results showed that nitrate supplementation increased the expression of *ALP*, *Run-2* and *OPN* in BMSCs of the OVX group. Data are presented as mean ± SD, n = 5. (For interpretation of the references to color in this figure legend, the reader is referred to the Web version of this article.)

To investigate whether nitrate exerts a preventive effect against osteoporosis by regulating the function of BMSCs in mice, we evaluated the effects of increasing nitrate on BMSCs in OVX mice. When BMSCs were cultured *in vitro*, Ki67 fluorescence staining showed that the number of Ki67-positive cells (Ki67+) was significantly lower in the BMSCs of the OVX group, while supplementation with 4 mM nitrate significantly increased it (Fig. 5A). Furthermore, flow cytometry analysis showed that the rate of ki67-positive cells in BMSCs of the 4 mM Nitrate group was higher than that in BMSCs of the OVX group (Fig. 5B), indicating that nitrate supplementation attenuated the decrease in proliferation rate of BMSCs in OVX mice *in vivo*. Alizarin red staining and quantitative analysis showed that the production of mineralized nodules in BMSCs of OVX group was less than that of Sham group, but supplementation with 4 mM nitrate significantly increased the production of mineralized nodules in BMSCs of OVX mice (Fig. 5C). Consistent with the phenotype, the mRNA expression levels of osteogenesis-related genes *ALP*, *Run-2* and *OPN* were down-regulated in BMSCs of the OVX group compared with the Sham group, while the mRNA expression levels of these indicators were significantly up-regulated in BMSCs of the 4 mM Nitrate group compared with the OVX group (Fig. 5D–F). The above results indicated that nitrate could enhance the proliferation and osteogenic differentiation potential of BMSCs in OVX mice.

### 3.6 Proteomic analysis of BMSCs in OVX mice regulated by nitrate

**Fig. 6:**
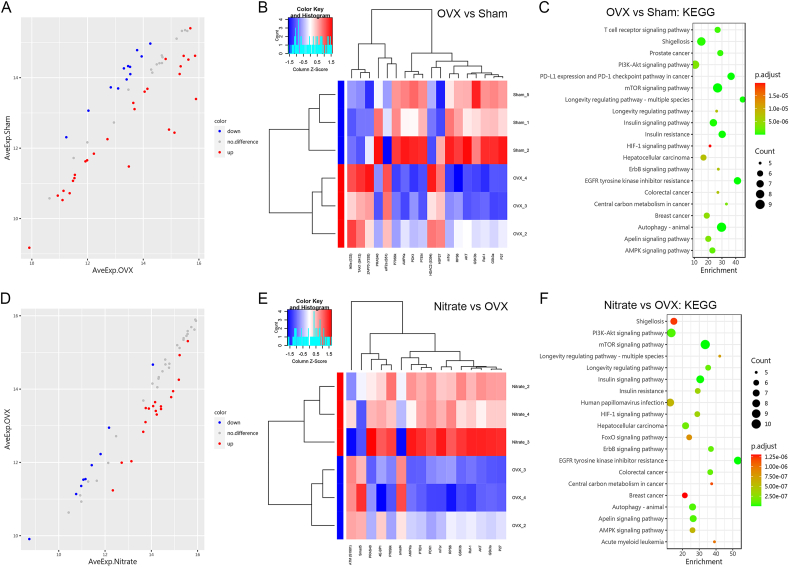
**Differentially expressed proteins of BMSCs in OVX mice regulated by nitrate.** A, The OVX group shows upregulated proteins in red, downregulated proteins in blue and non-differential proteins in grey compared to the Sham group. B, Cluster analysis of BMSCs from the OVX group and the Sham group to identify differentially expressed proteins. C, Scatter plot showing statistics of differentially expressed proteins between the OVX group and the Sham group analyzed by KEGG pathway enrichment. D, The 4 mM Nitrate group shows upregulated proteins in red, downregulated proteins in blue and non-differential proteins in grey compared to the OVX group. E, Cluster analysis of BMSCs from the 4 mM Nitrate group and the OVX group to identify differentially expressed proteins. F, Scatter plot showing statistics of differentially expressed proteins between the 4 mM Nitrate group and the OVX group analyzed by KEGG pathway enrichment. Data are presented as mean ± SD, n = 3. (For interpretation of the references to color in this figure legend, the reader is referred to the Web version of this article.)

**Fig. S1:**
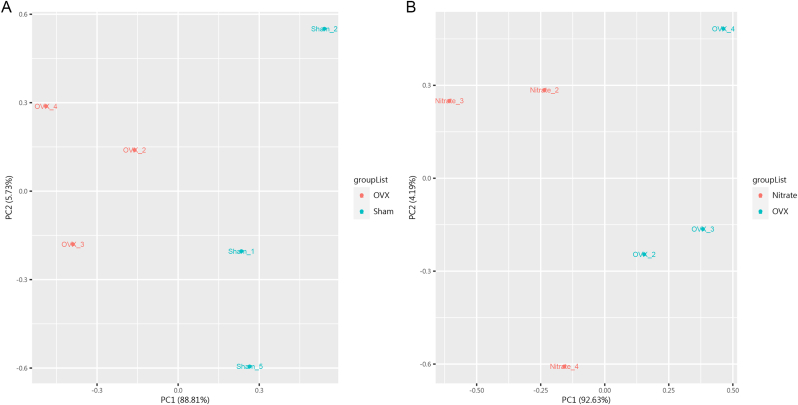
**Principal component analysis (PCA) in different groups**. (A) The OVX group versus the Sham group. (B) The 4 mM Nitrate group versus the OVX group.

**Fig. S2:**
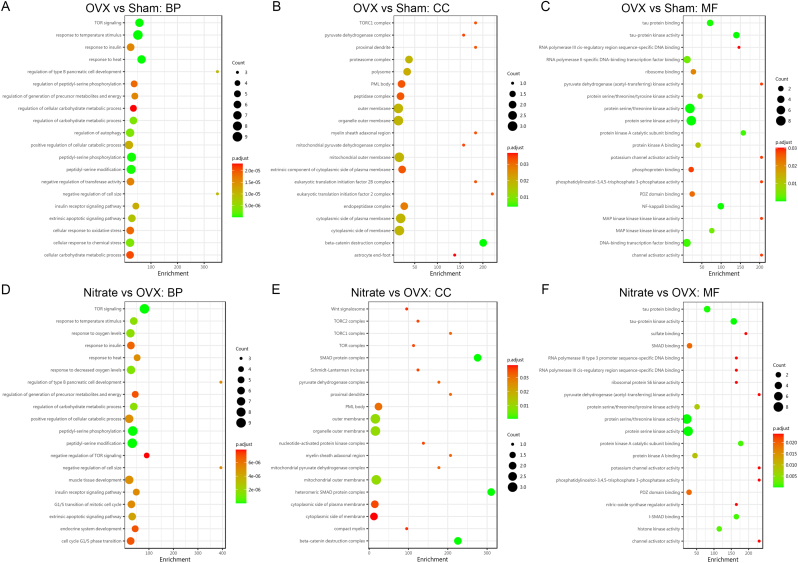
**Differentially expressed proteins in different groups of BMSCs on GO enrichment analysis**. (A) Scatter plot showed the statistical data of proteins in the OVX group versus the Sham group by biological processes enrichment analysis. (B) Scatter plot showed the statistical data of proteins in the OVX group versus the Sham group by cellular components enrichment analysis. (C) Scatter plot showed the statistical data of proteins in the OVX group versus the Sham group by molecular functions enrichment analysis. (D) Scatter plot showed the statistical data of proteins in the 4 mM Nitrate group versus the OVX group by biological processes enrichment analysis. (E) Scatter plot showed the statistical data of proteins in the 4 mM Nitrate group versus the OVX group by cellular components enrichment analysis. (F) Scatter plot showed the statistical data of proteins in the 4 mM Nitrate group versus the OVX group by molecular functions enrichment analysis.

Next, we analyzed proteins differentially expressed between the OVX group and the Sham group, and between the 4 mM Nitrate group and the OVX group, respectively, using proteomics techniques. Principal component analysis (PCA) was analyzed to observe the overall distribution trend among all the groups of samples (Fig. S1A, B). Differentially expressed proteins (DEPs) are defined as those with p-value (P.Val) less than 0.05, and fold change over 1.2 or less than 0.83, which are presented as Volcano plot and heatmap. There were 6 proteins upregulated and 12 proteins downregulated in the OVX group compared to the Sham group (Fig. 6A and B). There were 13 proteins upregulated and 3 proteins downregulated in the 4 mM Nitrate group compared to the OVX group (Fig. 6D and E). Scatter plot showed differential proteins in the OVX group versus the Sham group and the 4 mM Nitrate group versus the OVX group by GO enrichment analysis (Fig. S2A-F). The differential proteins were then labeled on the KEGG pathway graph and the results of the KEGG pathway enrichment analysis were displayed by scatter plots. Compared to the OVX group and the Sham group, DEPs were mainly enriched in signaling pathways such as EGFR tyrosine kinase inhibitor resistance, mTOR signaling pathway and longevity regulating pathway (Fig. 6C). Compared to the 4 mM Nitrate group and the OVX group, DEPs were mainly enriched in signaling pathways such as EGFR tyrosine kinase inhibitor resistance and mTOR signaling pathway (Fig. 6F).

### 3.7 Nitrate mediated osteoporosis prevention through EGFR-AKT-MAPK signaling pathway and mTOR-S6K signaling pathway

**Fig. 7:**
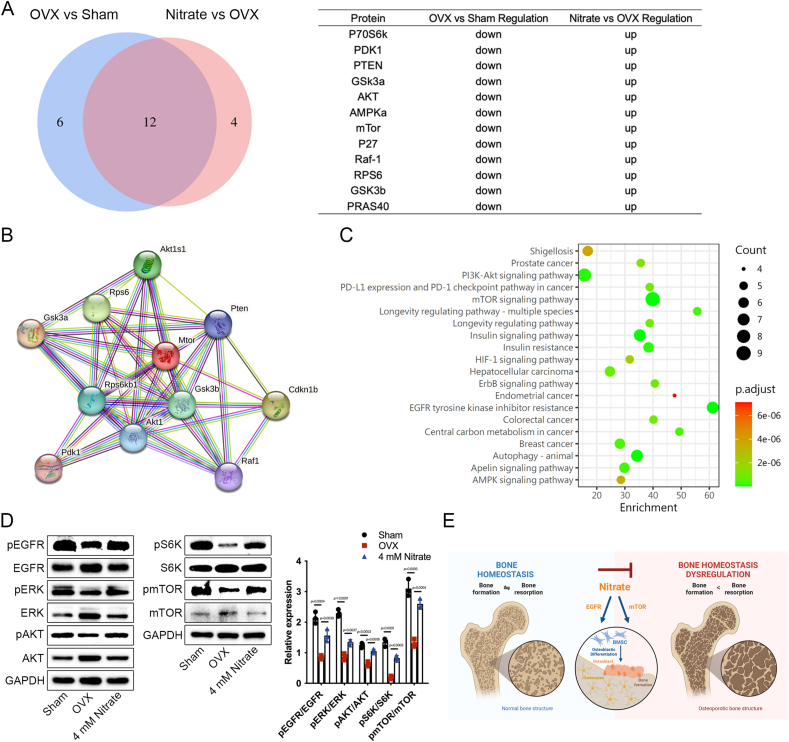
**Mechanism of nitrate regulation of BMSCs proliferation and osteogenic differentiation potential.** A, Venn diagram analysis showed that the overlap and unique different between proteins of BMSCs from different aged mice. B, PPI network map of overlapped differential proteins. C, Scatter plot showing the statistics of overlapping differentially expressed proteins analyzed by KEGG pathway enrichment. D, Nitrate upregulated phosphorylation of EGFR, ERK, AKT, mTOR and S6K in BMSCs of OVX mice. E, Schematic diagram of nitrate for prevention of osteoporosis. Created with BioRender. Data are presented as mean ± SD, n = 3.

Moreover, we compared the differentially expressed proteins by Venn diagram analysis, and 12 proteins were overlapped (Fig. 7A). We obtained a PPI network map of the overlapped parts 12 proteins (Fig. 7B). We then analyzed the function of the overlapped 12 proteins by KEGG pathway map. The data showed that the differences related to EGFR and mTOR signaling pathway were more significant (Fig. 7C). EGFR and mTOR were significantly upregulated in BMSCs of OVX mice after nitrate administration, suggesting activation of EGFR and mTOR signaling pathways (Fig. 7C). Extracellular regulatory protein kinase (ERK) and protein kinase B (AKT) are downstream effectors of the EGFR pathway and ribosomai protein S6 kinase (S6K) is a downstream effector of the mTOR pathway, which are involved in maintaining the homeostasis of BMSCs and promoting the proliferation and osteogenic differentiation potential of the cells. Nitrate administration increased the phosphorylation of EGFR, ERK and AKT in BMSCs of OVX mice, along with mTOR and S6K (Fig. 7D). These results suggested that nitrate could mediate cell proliferation and osteogenic differentiation through the EGFR-AKT-ERK signaling pathway and the mTOR-S6K signaling pathway to prevent osteoporosis (Fig. 7E).

## 4 Discussion

Osteoporosis is a generalized disorder of bone metabolism characterized by damage to the microstructure of bone tissue, decreasing ratios of bone mineral components and bone matrix, thinning of bone, reduction in the number of bone trabeculae, increase in bone brittleness, and increased risk of fracture [20]. Previous studies have found that dietary nitrates are absorbed into the bloodstream via the intestinal mucosa and later actively absorbed and secreted into the saliva by Sialin, the nitrate transport channel of salivary gland cells, which can play an important role in systemic health and disease prevention [21]. One study found improved BMD in postmenopausal women using organic nitrates [22], but another showed that neither long-term nitrate use nor duration of treatment was associated with lower fracture risk [23]. Therefore, further clarification is still needed on whether nitrates can be used as a preventive agent against osteoporosis by altering bone metabolism. BMSCs, as a major cell type for bone formation, and promotion of their osteogenic differentiation potential may be a promising strategy for bone formation [24]. The present study showed that salivary nitrate promotes the proliferation and osteogenic differentiation of BMSCs through the EGFR and mTOR signaling pathways, thereby preventing osteoporosis.

Bones have a certain amount of bone tissue being resorbed every day, and a considerable amount of bone tissue is synthesized, constantly replacing the old bone by the new one, and this process is bone metabolism [25]. Among the main etiologic factors of osteoporosis is impaired bone formation [26]. In this study, Micro-CT analysis and histological staining revealed that removal of salivary nitrate further aggravated the degree of bone tissue lesions in OVX mice, but administration of nitrate significantly prevented the osteoporotic phenotype in OVX mice. In addition, serum PINP, a product of type I collagen synthesis by osteoblasts, is an indicator of bone synthesis [27]. Further ELISA assays revealed that removal of salivary nitrate further decreased PINP levels in OVX mice, but administration of nitrate significantly elevated PINP levels in OVX mice. Our results suggest that salivary nitrate deficiency induces osteoporosis in the distal femur of mice, whereas nitrate supplementation produces a strong osteogenic effect on the femur of OVX mice, thereby maintaining bone homeostasis. This is consistent with previous observational cohort studies of organic nitrate as a nitric oxide donor in the treatment of osteoporosis [28], but we are prophylactic administration of inorganic nitrate, so there may be other mechanisms of action.

The function of BMSCs directly affects the number and activity of osteoblasts, which in turn regulates the synthesis, secretion and mineralization of bone matrix [29]. In our study, administration of nitrate significantly promoted the *in vitro* and *in vivo* proliferative capacity of BMSCs in OVX mice. The presence of nitrate enhanced the osteogenic differentiation potential of BMSCs in OVX mice by alizarin red staining, indicating that nitrate has a strong osteogenic effect on BMSCs of OVX mice. In addition, further PCR results revealed that nitrate significantly increased the expression levels of osteogenesis-related genes (*ALP*, *Run2* and *OPN*) in BMSCs of OVX mice. ALP is a key protein for early bone formation [30]. Run2 is an important osteogenic transcription factor responsible for the proliferation and differentiation of early osteoblasts [31]. OPN is a key component of the osteoclasts-produced bone one of the more abundant non-collagenous proteins in the matrix and is involved in bone formation and remodeling [32]. Our results suggest that nitrate promotes bone formation possibly by promoting the proliferation and osteogenic differentiation potential of BMSCs, thereby preventing osteoporosis.

To further clarify the underlying mechanisms, this study was conducted by means of proteomics in order to identify proteins and related pathways that may play a key role in promoting the proliferative and osteogenic differentiation potential of BMSCs during nitrate prevention of osteoporosis. The results identified 12 differential proteins that were common to the OVX group over the Sham group and the nitrate group over the OVX group and constructed interaction networks. Further KEGG analysis revealed that these 12 differential proteins were mainly enriched in EGFR and mTOR signaling pathways. Epidermal growth factor receptor (EGFR) signaling has been reported to be critical for bone metabolism, especially for the homeostatic maintenance of bone marrow mesenchymal stem cell function and new bone formation [33,34]. Inactivation of EGFR in mice by EGFR-specific inhibitors or knockdown of EGFR in osteoblasts and osteoclasts leads to a decrease in the number of bone marrow MSCs, which results in bone loss [35]. In line with this, our study found that EGFR/AKT/ERK signaling was down-regulated in BMSCs expressed in OVX mice, but significantly up-regulated by the administration of nitrate. In addition, mTOR signaling has an important role in regulating cell proliferation and osteogenic differentiation [36,37]. Our results revealed that mTOR/S6K signaling was down-regulated in BMSCs from OVX mice, but significantly up-regulated by the administration of nitrate. It is concluded that nitrate has a role in promoting bone formation and may be a candidate for osteoporosis.

Our results revealed new insights into the role of nitrate in the progression of osteoporosis and provided new therapeutic targets for osteoporosis treatment. These findings suggested that salivary nitrate levels might serve as a biomarker for osteoporosis, and that administration of nitrate significantly promoted bone formation, which could help to achieve early detection and prevention of the disease, with significant implications for reducing the prevalence of the disease.

## 5 Conclusion

In conclusion, we found that salivary nitrate could enhance the proliferation and osteogenic differentiation potential of BMSCs through the EGFR/AKT/ERK and mTOR/S6K signaling pathways, promote bone formation and prevent osteoporosis. These findings suggest that salivary nitrates may be a promising therapeutic target to maintain bone homeostasis and have wide clinical applications.

## Author contributions

SW and XW conceived and designed the experiments. XL and LeiH performed the experiments. XW, LuanH, JW and CZ assisted the experiments. XL analyzed the data. XL wrote the paper. SW and LeiH revised the manuscript. All authors read and approved the manuscript.

## Funding

This study was supported by the 10.13039/501100001809National Natural Science Foundation of China (82030031, 92149301, 91649124, 82350003, 92049201), 10.13039/501100005150Chinese Academy of Medical Sciences Research Unit (2019–12M-5-031), 10.13039/501100009592Beijing Municipal Science & Technology Commission No (Z181100001718208), Beijing Municipality Government grants (Beijing Laboratory of Oral Health-PXM2021_014226_000041; Beijing Scholar Program-PXM2021_014226_000021), Innovation Research Team Project of Beijing Stomatological Hospital, 10.13039/501100002799Capital Medical University (CXTD202201), Scientific Research Common Program of 10.13039/501100002888Beijing Municipal Commission of Education (KM202110025009) and 10.13039/501100005089Beijing Municipal Natural Science Foundation (7232071), Young Scientist Program of Beijing Stomatological Hospital, 10.13039/501100002799Capital Medical University (YSP202308).

## Declaration of competing interest

The authors declare no potential conflicts of interest with respect to the authorship and/or publication of this article.

## Appendix A Supplementary data

Supplemental file 2: Fig. S1. Principal component analysis (PCA) in different groups.

Supplemental file 3: Fig. S2. Differentially expressed proteins in different groups of BMSCs on PCA analysis.
