# Supplementary material for: Salivary nitrate prevents osteoporosis via regulating bone marrow mesenchymal stem cells proliferation and differentiation
Source: J Orthop Translat. 2024 Mar 25;45:188–96. doi: 10.1016/j.jot.2023.12.001 (PMC10982545; doi:10.1016/j.jot.2023.12.001)
Supplement: Multimedia component 1 [file mmc1.docx]

**Supplemental file 1**

**Table S1** **Gene primers**

| Gene Names | Forward/  Reverse | Sequences |
| --- | --- | --- |
| *GAPDH* | F | 5’-AGGAGAGTGTTTCCTCGTCC-3’ |
|  | R | 5’-TGCCGTGAGTGGAGTCATAC-3’ |
| *ALP* | F | 5’-GGTCACAGCAGTTGGTAGCTT-3’ |
|  | R | 5’-AATTGACGTTCCGATCCTGAGTG-3’ |
| *Runx-2* | F | 5’-TCGGAGAGGTACCAGATGGG-3’ |
|  | R | 5’-AGGTGAAACTCTTGCCTCGT-3’ |
| *OPN* | F | 5’-AAGCATCCTTGCTTGGGTTTG-3’ |
|  | R | 5’-ATGGTCGTAGTTAGTCCCTCAGA-3’ |

ALP: alkaline phosphatase; Runx2: runt-related transcription factor 2; OPN: osteopontin; GAPDH: glyceraldehyde 3-phosphate dehydrogenase.
